# Vital Statistics, Etc., and the Hygiene of the Convicts, Hospital, and Prison Camps of the Penitentiary of Georgia, from October 1, 1890, to October 1, 1894*Read at the meeting of the Georgia Medical Association, Savannah, April, 1895.

**Published:** 1895-08

**Authors:** W. O’Daniel

**Affiliations:** Bullard, Ga., Chief Physician to the Penitentiary


### VITAL STATISTICS, ETC., AND THE HYGIENE OF THE CONVICTS, HOSPITAL, AND PRISON CAMPS OF THE PENITENTIARY OF GEORGIA, FROM OCTOBER 1, 1890, TO OCTOBER 1, 1894.*

BY W. O'DANIEL, M.D., BULLARD, GA.,
Chief Physician to the Penitentiary.

As an appointee of Governor W. J. Northen, I assumed the official duties of Principal Physician to the Penitentiary of Georgia on the 1st day of April, 1891, for the four years ensuing; consequently my term expired April 1, 1895. For convenience, however, we will consider the medical department of the penitentiary covering the period from 1890 to 1894.

The first six months of this period my distinguished predecessor, Dr. H. V. M. Miller, was at the head of the department, he having consented to fill the unexpired term of the lamented Dr. W. F. Westmoreland, whose death caused the vacancy.

In Georgia, all of the convicts are owned and controlled by lessees, and are now worked in seventeen different counties in the State, viz: Dade, Walker, Bartow, Fulton, Oglethorpe, Elbert, Emanuel, Bulloch, Tattnall, Dodge, Wilcox, Dooly, Worth, Lowndes, Clinch, Charlton, and Ware. The penitentiary camps are, therefore, widely separated, and the prisoners confined therein

---

*Read at the meeting of the Georgia Medical Association, Savannah, April, 1895.

are necessarily exposed to varieties of location and climate, and they are compelled to perform various kinds of labor, such as iron and coal mining, manufacturing brick, agriculture, the lumber business, getting cross-ties for railroads, and producing naval stores. From 1890 to 1892 the total number of convicts handled by the penitentiary authorities was 2,802. Out of this number, during the *two* years, 107 went to "that undiscovered country from whose bourne no traveler returns."

From 1892 to 1893 the total number handled was 2,656, out of which number, during the year, 62 lost their lives.

From 1893 to 1894, 2,813 were controlled, and out of this number, from all causes, 63 died.

During the four years (from October 1, 1890, to October 1, 1894) 8,271 prisoners were handled by the penitentiary authorities. Out of this number during that time 232 deaths were reported from all causes. About 142 of these were from accidental injuries and *other* causes not amenable to hygiene and sanitary prevention. Approximately, 90 died from so-called preventable diseases.

The number of cases of *actual* sickness from 1890 to 1892 were 1,003 (for two years); from 1892 to 1893 were 697 (for one year); from 1893 to 1894 were 883; making the total number of cases of *all* kinds 2,583. The number killed by guards (trying to escape), by falling timbers, premature explosions in blasting, by cars, in mutiny, one executed by the law, and those who died from internal injuries, old age, and those who came from jails infected with gonorrhea and syphilis and their sequels, and from pulmonary consumption, heart disease, apoplexy, epidemic contagious diseases, epidemic la grippe, make about 950 cases during the four years, which, if deducted from 2,583 cases of all kinds during the four years, leaves 1,633 cases *possibly* preventable by perfect hygiene and faultless sanitary conditions, which to obtain in convict camps is a physical impossibility.

The number killed from 1890 to 1892:

| | |
|---|---|
| By premature blast explosion | 8 |
| In mutiny | 5 |
| Railroad cars | 1 |
| Slate fall | 1 |
| Executed for murder | 1 |
| Trying to escape | 2 |
| | 18 |

The number killed from 1892 to 1893:

| | |
|---|---|
| By premature blast explosion | 2 |
| Trying to escape (shot) | 3 |
| Falling timbers | 2 |
| By railroad cars | 1 |
| | 8 |

The number killed from 1893 to 1894:

| | |
|---|---|
| By slate fall | 1 |
| By railroad cars | 1 |
| By guard for resisting | 1 |
| By falling timbers | 3 |
| By trying to escape | 3 |
| By lightning | 1 |
| | 10 |

Making a total of 36 killed outright in the four years. Of the 232 deaths in the four years from all causes, 36 were killed in the way stated above; about 106 from causes and diseases non-preventable *in prison camps;* about 90 from so-called preventable diseases.

From all causes the death-rate was *about* 35 in the thousand.

From diseases possibly remediable or preventable *about* 13 in the thousand per annum, or $1\frac{3}{10}$ per cent. This is a low death-rate, when we consider the fact that convicts come to the penitentiary directly from the jails; many of them after long confinement, and quite a number already suffering from chronic and constitutional diseases, which would be incurable under most favorable circumstances and the best hygienic and sanitary surroundings and conditions, which we know cannot exist in penitentiary camps. Therefore, our tables of sickness and mortality have not yet been reduced as low as we would have them, but four years' experience in the Medical Department of the Georgia Penitentiary convinces me that ceaseless care and the frequent visitation and inspection of camps and the strictest observance of progressive hygiene and sanitation will reduce *preventable* sickness and mortality to the lowest possible minimum.

Old age, chronic and constitutional diseases, will unavoidably continue to increase the death-rate, so as to apparently make it widely disproportionate, to the number *actually* sick in hospitals.

During the four years about twenty deaths were reported from typhoid fever. Nearly all of them were from the northern part of the State, only two cases, I believe, from the southern or wire-grass region. Genuine typhoid fever, or typical cases of typhoid fever, are seldom seen in the pine region of Georgia. We have what our physicians choose to call typho-malarial fever, or continued. Dr. K. H. Davis, who has lived and practiced medicine in the mountains of North Georgia forty years I suppose, says in a letter to me: "This death-dealing disease does not regard the high, or the low. It is found as often in the mansion as in the hovel. I think the cause of it is from polluted water, which we drink, made so by the fermentation of fecal matter, hog pens, bad sewerage, decomposition of vegetable and animal matter—fine soil for this death-dealing microbe." It is only just to say, that the camps where this disease prevailed mostly were what are called "home camps," or receiving and distributing hospitals for the penitentiary; therefore, more invalids and convalescents were to be found at these North Georgia camps.

In the four years, about thirty deaths have been reported from pulmonary consumption; those engaged in coal-mining most susceptible, *especially* when there was an heredity or predisposition.

Georgia ought to be an inviting field for our Northern friends, who are health-seekers, and especially to those who suspect pulmonary predispositions and inheritance. The pine region of Georgia is equal to Colorado for the consumptive. We have not the altitude, but we have the terebinthinates in abundance, the very best remedial agent for the mucous membranes of the bronchi.

The convicts in the Georgia penitentiary, as a rule, enjoy greater immunity from preventable diseases than free people in neighborhoods adjacent to the prisons. This ought to satisfactorily demonstrate the fact that sickness and the death-rate, when caused by diseases amenable to sanitary conditions, decrease proportionately with the improved methods of sanitation.

Our methods of hygiene and general sanitation in prison camps must necessarily be crude and imperfect, but we constantly urge and require scrupulous cleanliness of the buildings in which the well are kept, and of the hospitals, as well as personal hygiene.

The water supply for drinking purposes must receive special and unceasing attention. He who imbibes polluted water unsuspectingly endangers his health and his life.

Personal hygiene and regularity of habits play a full hand in the prevention of diseases; and he who intelligently prevents disease, pain, and death is more deserving than he who skillfully cures; hence, the oft-repeated adage: "An ounce of prevention is worth a pound of cure."

8,271 convicts controlled by penitentiary authorities in Georgia, from October 1, 1890, to October 1, 1894.

232 of that number lost their lives from all causes, during that period.

36 were killed outright, in ways before stated in this paper, during the four years.

106 died from diseases and other causes not amenable to hygienic and sanitary prevention.

90 from so-called preventable diseases.

35 *in the thousand* lost their lives from *all* causes.

13 *in the thousand*, from diseases thought to be preventable.

Or, a $1\frac{3}{10}$ per cent. death-rate.

